# Training on an Appetitive Trace-Conditioning Task Increases Adult Hippocampal Neurogenesis and the Expression of Arc, Erk and CREB Proteins in the Dorsal Hippocampus

**DOI:** 10.3389/fncel.2020.00089

**Published:** 2020-04-17

**Authors:** Shweta Tripathi, Anita Verma, Sushil K. Jha

**Affiliations:** School of Life Science, Jawaharlal Nehru University, New Delhi, India

**Keywords:** appetitive conditioning, cell proliferation, hippocampal neurogenesis, trace, delay conditioning

## Abstract

Adult hippocampal neurogenesis (AHN) plays an essential role in hippocampal-dependent memory consolidation. Increased neurogenesis enhances learning, whereas its ablation causes memory impairment. In contrast, few reports suggest that neurogenesis reduces after learning. Although the interest in exploring the role of adult neurogenesis in learning has been growing, the evidence is still limited. The role of the trace- and delay-appetitive-conditioning on AHN and its underlying mechanism are not known. The consolidation of trace-conditioned memory requires the hippocampus, but delay-conditioning does not. Moreover, the dorsal hippocampus (DH) and ventral hippocampus (VH) may have a differential role in these two conditioning paradigms. Here, we have investigated the changes in: (A) hippocampal cell proliferation and their progression towards neuronal lineage; and (B) expression of Arc, Erk1, Erk2, and CREB proteins in the DH and VH after trace- and delay-conditioning in the rat. The number of newly generated cells significantly increased in the trace-conditioned but did not change in the delay-conditioned animals compared to the control group. Similarly, the expression of Arc protein significantly increased in the DH but not in the VH after trace-conditioning. Nonetheless, it remains unaltered in the delay-conditioned group. The expression of pErk1, pErk2, and pCREB also increased in the DH after trace-conditioning. Whereas, the expression of only pErk1 pErk2 and pCREB proteins increased in the VH after delay-conditioning. Our results suggest that appetitive trace-conditioning enhances AHN. The increased DH neuronal activation and pErk1, pErk2, and pCREB in the DH may be playing an essential role in learning-induced cell-proliferation after appetitive trace-conditioning.

## Introduction

New neurons are continuously generated from multipotent neural stem cells (NSCs) in the adult brain in the subventricular zone (SVZ) of the forebrain and subgranular zone (SGZ) of the dentate gyrus (DG) of the hippocampus (Lledo et al., 2006; Deng et al., 2010; Ming and Song, 2011; Urbán and Guillemot, 2014; Shohayeb et al., 2018). Several reports suggest the role of adult hippocampal neurogenesis (AHN) in learning and memory associated with various hippocampus-dependent/independent tasks. Furthermore, previous studies have shown that the hippocampus-dependent but not independent tasks may modulate AHN (Gould et al., 1999; van Praag et al., 1999; Snyder et al., 2005; Epp et al., 2007; Kitamura et al., 2009; Deng et al., 2010; Castilla-Ortega et al., 2011; Marín-Burgin and Schinder, 2012). Recent studies regarding the functionality of these newly born cells in the SGZ of the hippocampus have shown that newborn neurons are specifically important for the formation and long term persistence of hippocampus-dependent memory (Epp et al., 2007; Dupret et al., 2008; Sahay et al., 2011; Pan et al., 2012a,b, 2013; Kitamura and Inokuchi, 2014; Abrous and Wojtowicz, 2015). Nevertheless, the effects of learning on adult neurogenesis and conversely the role of neurogenesis in the facilitation of learning and memory are not known.

Many groups have attempted to describe the underlying molecular mechanisms for learning-induced AHN. It has been found that calcium response element-binding protein (CREB), a family of transcription factors, plays an essential role in memory processing by enhancing AHN (Silva et al., 1998; Impey et al., 1998a,b; Stanciu et al., 2001; Desmedt et al., 2003; Ortega-Martínez, 2015). The increased CREB signaling in the hippocampus promotes cell proliferation and survival of newly born neuronal cells (Nakagawa et al., 2002; Fujioka et al., 2004; Merz et al., 2011). Extracellular signal-regulated kinases (Erks), an upstream regulator of CREB activation cascade, have also been reported to induce cell proliferation and differentiation (Lefloch et al., 2009; Oliveros et al., 2017). Besides, Erks and CREB, and activity-regulated cytoskeleton-associated protein (Arc), have also been shown to be up-regulated in response to hippocampal neuronal activation (Lyford et al., 1995; Waltereit et al., 2001; Ying et al., 2002). Studies have also shown that the dorsal hippocampus (DH) and ventral hippocampus (VH) play a differential role in the consolidation of different memory types. For example, spatial memory was found to be explicitly dependent on the DH but not on the VH (Moser et al., 1993, 1995). Moreover, selective VH lesions resulted in altered stress responses and emotional behaviors but spared spatial memory (Moser et al., 1993, 1995). However, functional dissociation of the DH and VH in associative conditioning tasks has not been explored in detail.

Spatial learning tasks, such as Morris water maze, are the most extensively used paradigm to study the role of AHN in learning and memory. There are some reports where associative conditioning models such as contextual/cued fear conditioning have also been used to study their effects on neurogenesis and in turn how AHN may modulate hippocampus-dependent or independent learning/memory (Leuner et al., 2006; Saxe et al., 2006; Kitamura et al., 2009; Kim et al., 2012; Marín-Burgin and Schinder, 2012; Seo et al., 2015; Ishikawa et al., 2016). However, in these studies, the effects of the stressful events that animal has to face during such aversive or anxiogenic form of training are also accompanied (Baron and Galizio, 2005; Lucassen et al., 2015).

Appetitive conditioning is another form of associative conditioning, where an individual learns to predict the possible outcomes of the conditioned stimulus (CS) by extracting the logical information after repeated exposure (Tripathi and Jha, 2016; Tripathi et al., 2018, 2019). Appetitive conditioning can be further classified as non-aversive or aversive forms, based on the motivational or hostile environment that the animal has to face to obtain food (Tripathi et al., 2019). Recent findings have determined the role of the hippocampus in fear as well as appetitive memory (Sanders et al., 2003; Hernández-Rabaza et al., 2008; Reichelt and Lee, 2013; Kant and Jha, 2019). Also, it has been reported that CREB, Erks, and Arc proteins play an essential role in both fear and appetitive memories (Trifilieff et al., 2006; Johansen et al., 2011; Isokawa, 2012; Tronson et al., 2012). However, it is not known if the appetitive conditioning task can also influence AHN in the hippocampus. Further, if the DH and VH play a differential role in the appetitive trace- and delay-conditioning is also not well-known. In our study, we have used the trace- and delay-conditioning forms of classical appetitive conditioning to evaluate its effect on cell proliferation in the DG area of the hippocampus and the progression of these proliferative cells towards the neuronal lineage in the adult brain. Also, we have investigated the expression/activation of CREB, Erk1, and Erk2 and Arc proteins in the DH and VH, to study the specific role of the DH and VH in the appetitive trace- and delay-conditioning induced cell proliferation.

## Materials and Methods

### Animals

Male *Wistar* rats (200–230 g, *N* = 73) were used for the study. Animals were brought from the University’s Central Laboratory Animal Research (CLAR) facility to our institutional animal house. They were housed in their home cages for 1 week before the commencement of experiments. During this period, animals were maintained on a 12–12 h Dark/Light cycle (lights on at 7:00 AM) at 23–24°C temperature, and food and water were provided *ad libitum*. All experimental procedures were approved by the Institutional Animal Ethics Committee (IAEC protocol#17/2012), Jawaharlal Nehru University, New Delhi.

We have conducted two sets of experiments: Experiment-I: to investigate the influence of trace- and delay-conditioning on cell proliferation (*n* = 37) and the progression of these proliferative cells towards the neuronal lineage (*n* = 18) and Experiment-II: to investigate the changes in the expression levels of Erk1, Erk2, CREB, and Arc protein after trace- and delay-conditioning (*n* = 18). In both the experiments, animals were randomly divided into three groups: (i) trace-conditioned group; (ii) delay-conditioned group; and (iii) un-conditioned control group. The animals in the trace- and delay-conditioned group were trained for their respective appetitive conditioning tasks. However, the animals in the un-conditioned control group were not trained for any appetitive task; instead, they remained in their home cages for the entire experimental period.

### Appetitive Conditioning

Animals were trained for appetitive conditioning task in a conditioning chamber (12” × 12” × 11”). The conditioning chamber was kept inside a well-ventilated, sound and light dampened, dark-colored plexiglass behavioral chamber (4’ × 2’ × 2’), to minimize the external disturbances during the experiments. In the behavioral chamber, diffused light (20 Lux) was maintained continuously during the experiments. During appetitive conditioning, house light (fitted on the roof of conditioning chamber) was used as the CS, and mango fruit juice (Tropicana product, Pepsico India) was used as the unconditioned stimulus (US). The CS and US were presented through a computer using Graphic State software (Coulborn, Inc., Whitehall, PA, USA).

During conditioning, fruit juice was delivered through a liquid dipper set-up (Coulborn, Inc., Whitehall, PA, USA). One end of the lever of liquid dipper unit was connected to a computer controlled-motor, whereas, a small food cup was attached at the other end. The liquid dipper cup carried approximately 100 μl juice from the juice tray to the dispensing window. Animals had to poke their heads in the dispensing window to get the fruit juice. Number of head entries into the juice dispensing window (an outcome measure of learning) was registered in the computer through the Graphic State software (Coulborn, Inc., Whitehall, PA, USA) using a photo beam sensor, attached on the sidewalls of juice dispensing window.

**Figure 1:**
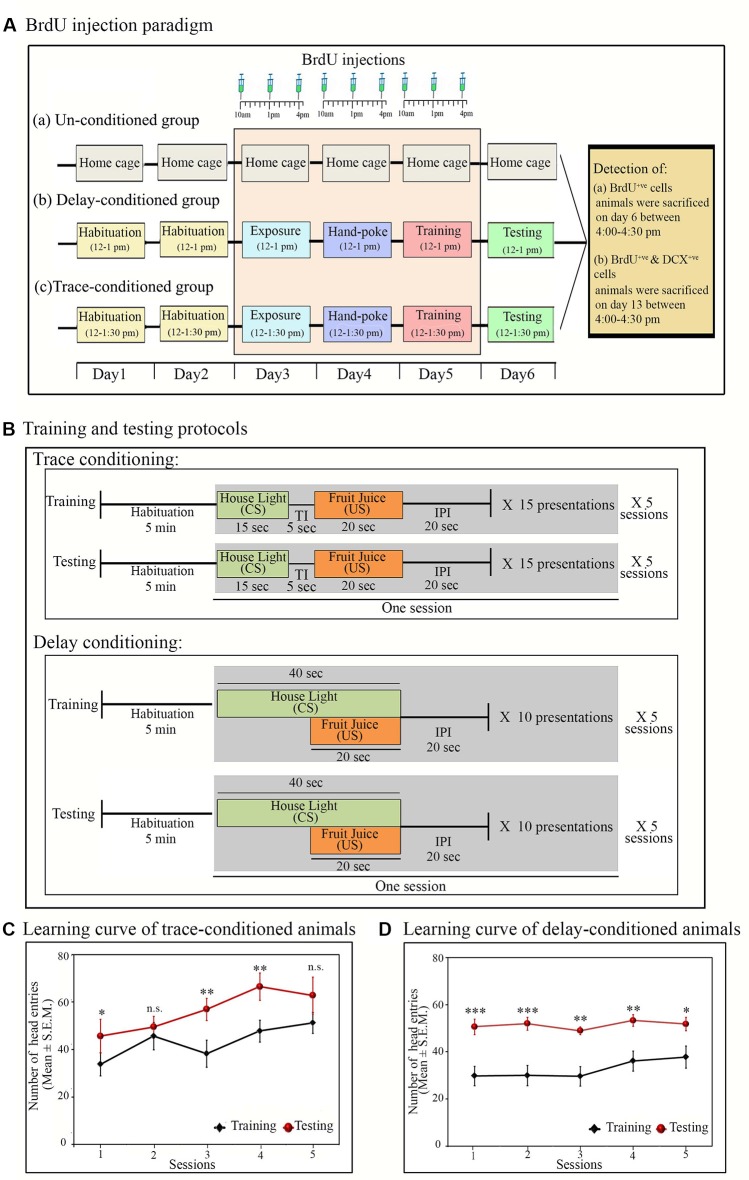
Experimental protocols: **(A)** time and days of BrdU (100 mg/kg) injection in (a) un-conditioned, (b) delay-conditioned and (c) trace-conditioned groups. BrdU was injected three times a day at an interval of 3 h, for three consecutive days (from day 3 to day 5) in all groups. In the conditioned groups: the first injection was given 2 h before the exposure/training, the second injection was given immediately after the completion of exposure/training, and the third injection was given 3 h after the exposure/training. In un-conditioned controls, no training was performed, but BrdU injection was given at the time-matched hours. Conditioned animals were tested for their learned tasks the next day at the same time and sacrificed 3 h after the testing. un-conditioned animals were sacrificed at the time matchedhours of trace/delay conditioned animals. For quantification of DCX^+ve^ cells, the animals were sacrificed on Day 13 (7 days after appetitive-conditioned training) at time-matched hour. **(B)** Trace- and delay-conditioning protocols: training for trace-conditioning was performed in five sessions. During training, after 5 min of habituation, house light was presented for 20 s as the conditioned stimulus (CS) followed by a trace interval (TI) of 5 s. After trace interval (TI), fruit juice was presented for 15 s as the unconditioned stimulus (US), which is then followed by an inter presentation interval (IPI) of 20 s. Thus, the entire presentation period was of 60-sec duration. Fifteen such presentations were repeated during one session. Complete training comprised of a total of 75 CS and US presentations. The next day, testing was performed at the time matched hour. The testing protocol was similar to that of training. Delay-conditioning: training was performed in five sessions. After 5 min of habituation, house light was presented as CS for 40 s. After 20 s of CS onset, fruit juice was presented as the US for 20 s. Both the CS and US were co-terminated at the end of 40 s, followed by 20-s IPI. The entire presentation was of 60-s duration. Ten such CS-US pairs were presented in one session. Complete training comprised of a total of 60 CS-US presentations. The animal was tested the next day for the learned task using a similar protocol, as was presented during the training. **(C)** Learning curve of the trace- and **(D)** delay-conditioned animals. Animals showed an increased number of head entries across all sessions on the testing day during the US presentation phase in the trace-conditioning and during the CS-US paired presentation phase in the delay-conditioning. *p < 0.05, **p < 0.01 and ***p < 0.001, n.s., non-significant. One way ANOVA followed by Tukey post hoc.

For appetitive conditioning, the animal was first habituated in the conditioning chamber for two consecutive days (Day 1 and 2). On Day 3, the animal was again kept inside the conditioning chamber and was exposed to the fruit juice (Tropicana Mango) *via* a water bottle and food cup (exposure day). It was done to familiarize the animal with the aroma and taste of fruit juice. The next day (Day 4), during the habituation, the experimenter trained the animal to approach the juice dispensing window to obtain the fruit juice. The fruit juice was provided by manually lifting the lever of the liquid dipper (hand-poke training). The process was repeated 15–20 times so that the animal gets the precise idea that the fruit juice is only given through the juice dispensing window. The majority of the animals learned the location of the juice dispensing window after this small training. Those, which were not able to locate the window, were retrained the next day during the same period. On Day 5, the animal was finally trained for appetitive trace/delay conditioning tasks (Figure 1A).

### Trace-Appetitive Conditioning

In the appetitive trace-conditioning task, CS and US presentations were separated by a small-time lag of 5 s (trace-interval). Trace-conditioning protocol comprised of four phases: (i) CS alone; (ii) trace interval (TI); (iii) US alone; and (iv) inter-presentation interval (IPI). The training was performed in five sessions over some time of 1 h and 30 min (12:00 pm to 1:30 pm).

During the trace-conditioning, the animal was placed in the conditioning chamber for 5 min (habituation time before training session); thereafter, the CS (house light) was delivered for 15 s. After a gap of 5 s (TI), the US (fruit juice) was presented for 20 s. No stimulus was presented for the next 20 s (IPI). The entire presentation was of 1 min duration. In each session, 15 CS and US presentations were dispensed. The training was performed in five sessions with an inter-session interval of 2 min. During the conditioning, a total of 75 presentations of CS and US were dispensed (Figure 1B). After completion of training, the animal was left in the conditioning chamber for an additional 5 min and then transferred back to the home cage. The animal was tested for the learned task 24 h later (day 6). During the testing, the animal was placed inside the conditioning chamber, and the CS and US were presented similarly, as were presented during the training (Figure 1B).

### Delay-Appetitive Conditioning

In delay-appetitive conditioning, CS and US were presented in a paired fashion. The delay-conditioning protocol comprised three phases: (i) CS alone; (ii) CS-US paired presentation; and (iii) IPI. The training was performed in five sessions, over some time of 1 h (12:00 pm to 1:00 pm).

During the delay-conditioning, the animal was placed in the conditioning chamber for 5 min for initial habituation. Thereafter, the CS (house light) was delivered for 40 s, which co-terminated with 20 s of US (fruit juice) presentation followed by an IPI of 20 s. The entire presentation was of 1 min duration. In each session, 10 CS-US paired stimuli were presented. The training was performed in five sessions with 2 min of inter-session interval. A total of 50 CS-US paired stimuli were presented during the conditioning (Figure 1B). The animal was returned to their home cages 5 min after the completion of the training trials. The animal was tested for the learned task 24 h later (day 6). During the testing, the CS-US pair was presented similarly as were presented during the training period (Figure 1B).

### BrdU Administration

BrdU (100 mg/kg, Sigma Aldrich) was dissolved in sterile saline solution (0.9%), and pH was brought to 7.4 by adding 1M NaOH solution. For cell proliferation study (Experiment-I), animals were injected BrdU intraperitoneally (i.p.), three times a day at an interval of 3 h for three consecutive days (Figure 1A). BrdU was injected 2 days before and on the appetitive-conditioned training day, and it was not injected on the testing day. The last injection of BrdU was made 3 h after conditioning. BrdU was injected in a total of 55 animals [trace-conditioned and DG cell-proliferation group (*n* = 10); delay-conditioned and DG cell-proliferation group (*n* = 9); trace-conditioned and SVZ cell proliferation group (*n* = 3); delay-conditioned and SVZ cell proliferation group (*n* = 3); un-conditioned and DG cell proliferation group (*n* = 9); un-conditioned and SVZ group (*n* = 3); trace-conditioned and neuronal lineage group (*n* = 6); delay-conditioned and neuronal lineage group (*n* = 6) and un-conditioned and neuronal lineage group (*n* = 6)]. The next day, all animals were tested for the learned tasks and sacrificed at a time-matched hour with the last BrdU injection (i.e., 3 h after testing; Figure 1). However, to trace the progression of proliferative cells towards neuronal lineage (DCX^+ve^ cells) after appetitive conditioning, the animals were sacrificed 7 days after appetitive conditioning (on day 13; Figure 1A).

### Immunohistochemistry

For the immunohistochemical examination of BrdU^+ve^, doublecortin^+ve^ (DCX^+ve^), and Ki67^+ve^ cells, the animal was perfused transcardially with 4% paraformaldehyde. The brain was extracted and kept in 4% paraformaldehyde overnight. Thereafter, it was kept in a 30% sucrose solution for 2 days. Once the brain completely submerged in the sucrose solution, histological sections were cut. A 30 μm thick coronal sections were cut on a cryostat (Thermo Fisher Scientific), and sections were stored overnight in 0.1 M PBS at 4°C.

On the following day, the sections were treated with 2N HCl at 50°C for 30 min on a shaker followed by washing with 0.1 M phosphate buffer saline (PBS; four times for 5 min each). After washing, sections were incubated in the blocking solution (4% Goat serum in 0.1 M PBS with 0.3% Triton-X) overnight at 4°C and then transferred to mouse anti-BrdU primary antibody (Sigma–Aldrich; 1:150, in blocking solution) for 48 h at 4°C on a shaker. After incubation with the primary antibody, sections were washed three times with 0.1 M PBS (5 min each) and then incubated in Alexa-488 anti-mouse antibody (1:800, Invitrogen) in 0.1 M PBS with 0.3% Triton-X for 24 h at 4°C in the dark. Sections were then washed with 0.1 M PBS (three times for 5 min each) and mounted on glass slides in fluoroshield mounting medium (Sigma–Aldrich) and stored in the dark at 4°C under moist conditions.

For BrdU^+ve^ and DCX^+ve^ double-labeling, 30 μm thick free-floating brain sections were treated in a similar way as mentioned above except that the sections were incubated together with two primary antibodies (in blocking solution): mouse anti-BrdU (1:150, Sigma–Aldrich) and rabbit anti-DCX (1:500, Abcam) for 24 h at 4°C. The sections were then washed with 0.1 M PBS (three times, 5 min each). Thereafter, sections were incubated in Alexa 488 tagged anti-mouse (1:800, Invitrogen) and Alexa 555 tagged anti-rabbit (1:1,000, Invitrogen) secondary antibodies in 0.1 M PBS with 0.3% Triton-X at 4°C for 24 h in dark. After secondary incubation, sections were again washed three times with 0.1 M PBS and mounted on glass slides in fluoroshield mounting medium (Sigma–Aldrich) and stored at 4°C in the dark for further microscopy analysis.

Also, we characterized the changes in cell-proliferation after appetitive conditioning using another effective mitotic marker “Ki67.” Although BrdU protocol is a gold standard to study cell proliferation, we used an antibody against Ki67 protein to characterized BrdU^+ve^ and Ki67^+ve^ double-labeled cells in nine animals (three animals/group). It was done to validate our BrdU results. For Ki67 and BrdU double-labeling, the brain sections were similarly processed for immunostaining as mentioned above except that the sections were incubated together in two primary antibodies: mouse anti-BrdU (1:150, Sigma–Aldrich) and rabbit anti-Ki67 antibody (1:400; Cell signaling technologies, India) in blocking solution for 48 h at 4°C. The sections were then washed with 0.1 M PBS for 5 min for three times. Thereafter the sections were incubated overnight in Alexa 488 tagged anti-mouse (1:800, Invitrogen) and Alexa 555 tagged anti-rabbit (1:1,000, Invitrogen) antibody in 0.1 M PBS with 0.3% Triton-X at 4°C. The sections were washed and mounted on glass slides in fluoroshield mounting medium and stored in the dark under moist conditions at 4°C.

### Western Blot

A separate group of animals (*n* = 18) were used for western blot experiments (trace-conditioned: *n* = 6; delay-conditioned: *n* = 6 and un-conditioned control: = 6). The trace- and delay-conditioned animals were sacrificed 1 h after the training, with xylazine (10 mg/kg) and ketamine (85 mg/kg) overdose, and the brain was extracted. The un-conditioned controls animals were also sacrificed similarly at a time-matched hour. The hippocampus was removed, and one side was kept intact, while the other side was further dissected into the DH and VH. The selection for keeping one side of the hippocampus intact and dissection of the other side was done randomly. The hippocampal tissue was dipped in the lysis buffer [RIPA (3 ml/gm), in which phenylmethyl sulfonyl fluoride (PMSF; 1 mM) and Protease Inhibitor Cocktail (PIC; 1:100)] were also added. The tissue was incubated for 10 min, and thereafter it was homogenized on ice. Tissue lysate was then centrifuged at 10,000 rpm for 30 min at 4°C. The supernatant was collected and stored at −80°C for further analysis.

Electrophoresis was performed as per the standard protocol. In brief, the total protein content in each sample was quantified using the Bradford assay. Samples for western blot were prepared. An equal amount of protein (50 μg per well) was loaded and resolved in 10% SDS-PAGE (BioRad western unit). Proteins were electro-blotted onto the PVDF membrane at 10V for 30 min (TransBlot semidry, BioRad). The membrane was blocked with 5% BSA in Tris-buffered saline (TBS) for 2 h at room temperature. After blocking, the membrane was washed with TBST (TBS with 0.5% Tween-20) and then incubated with primary antibody solution (in TBST) overnight at 4°C. After primary incubation, the membrane was washed with TBST (four times) and then incubated in secondary antibody for 3 h at room temperature. After washing, protein bands were visualized using the chemiluminescence method [ECL (Abbkines)] in ChemiDoc (Bio-Rad ChemiDoc TM XRS^+^ systems) using “Quantity One” software (Bio-Rad, USA).

Primary antibodies used for western blot analysis were, anti-CREB (1:1,000, Abcam), anti-pCREB (1:1,000, Abcam), anti-ErK1&2(1:1,000, Abcam), anti-pErk1&2 (1:1,000, Abcam), anti-Arc (1:2,000, Abcam), and anti β-actin (1:2,500, Sigma–Aldrich). Secondary antibodies used for the western blot analysis were as follows; goat anti-rabbit polyclonal HRP-tagged (1:10,000, Santa Cruz Biotechnology) and goat anti-mouse polyclonal HRP-tagged (1:10,000, Santa Cruz Biotechnology). The pre-stained protein ladder (Genedirex) was used for the identification of desired protein bands.

### Data Analysis

#### Trace- and Delay-Conditioning

The average number of head entries during the different phases on the training and testing days of the trace- and delay-conditioning tasks, were statistically analyzed using Sigma Stat 12.0 software (Systat, Inc. Chicago, IL, USA). The data was analyzed between groups using one-way analysis of variance (ANOVA) and within-group session-wise learning curve using one-way RM ANOVA followed by Tukey *post hoc* test. We have also calculated the values of variance, effect size, and power. It was done to demonstrate the spread of data and also the size of the effect.

#### Quantification of BrdU^+ve^ Cells, BrdU^+ve^ and DCX^+ve^ Double-Labeled Cells, and BrdU^+ve^ and Ki67^+ve^ Double-Labeled Cells

For the quantification of BrdU^+ve^, DCX^+ve^, and Ki67^+ve^ cells, immunostained sections were microscopically analyzed under 10× and 20× magnification using the Olympus BX53 epifluorescent microscope. The numbers of BrdU-labeled cells (green fluorescent dots) in the DG and its cross-sectional areas were counted using Image J software. A total of 10 hippocampal histological sections per animal (every 6th section, 180 μm apart, starting from the Bregma: −2.76 to −4.56 (Paxinos and Watson, 2009) were used for the counting of BrdU^+ve^ cells in the DG area. The number of BrdU^+ve^ cells per mm^3^ of DG, in each animal, was obtained by dividing the total number of BrdU^+ve^ cells by volume of DG. The volume of the DG was calculated using the standard formula V = T × (∑A_1-m_) where V = volume; T = distance between the sections [30 × 6 = 180 μm or 0.18 mm, in this case, A = area of DG in each section (A_1-m_ in our case is _1–10_; Dalla et al., 2009)]. The numbers of BrdU^+ve^ and Ki67 ^+ve^ cells in the DG and BrdU^+ve^ in the SVZ were also calculated similarly. However, in the SVZ cell-proliferation groups, we took a total of six sections/animal (every 4th section in each animal) and accordingly the volume of SVZ was calculated.

The average number of BrdU-labeled cells per mm^3^ in the DG and SVZ in the trace-conditioned, delay-conditioned and un-conditioned control animals were calculated and compared statistically using one-way ANOVA followed by Tukey *post hoc* test. We also calculated the values of variance, effect size, and power to demonstrate the spread of data and the size of the effect. Besides, the Pearson test was also performed to determine a correlation between the percent change in the number of proliferating cells and appetitive-conditioned learning.

To determine the progression of proliferative cells towards the neuronal lineage after appetitive conditioning, the immunostained sections of trace-conditioned, delay-conditioned, and un-conditioned control animals were microscopically analyzed under 20× magnification using Olympus BX53 fluorescent microscope. The number of BrdU^+ve^ cells (green fluorescent dots) and BrdU^+ve^ and DCX^+ve^ double-labeled cells (yellow or orange) in the DG area were counted using Image J software. A total of 10 histological sections per animal (every 6th section, 180 μm apart, starting from the Bregma: −2.76 to −4.56 (Paxinos and Watson, 2009) were used for the counting of BrdU^+ve^ and BrdU^+ve^ and DCX^+ve^ double-labeled cells in the DG area. The number of BrdU^+ve^ and BrdU^+ve^ and DCX^+ve^ double-labeled cells in the DG area of trace-conditioned, delay-conditioned, and un-conditioned control animals were calculated and compared statistically using one-way ANOVA followed by Tukey *post hoc* test.

#### Western Blot Analysis

All western blot images from the “Quantity One” software of Bio-Rad ChemiDoc TM XRS^+^ systems were used to determine the protein band intensity and relative changes in their expression level. We performed a densitometric analysis of blots using Image J software. The absolute intensity of Arc, CREB, pCREB, and Erk1, pErk1, Erk2 and pErk2 proteins blots were normalized with their respective loading control “β-actin” bands in each gel. The normalized values were calculated by dividing the band intensity of each protein with the band intensity of its corresponding β-actin blot. The relative changes in the expression level of Arc, CREB, pCREB, and Erk1, pErk1, Erk2, and pErk2 proteins in the dorsal, ventral, and total hippocampus were compared statistically between groups using one-way ANOVA followed by Tukey *post hoc* test. We further calculated the variance, effect size, and power values to demonstrate the spread of data and the size of the effect.

## Results

### Experiment-I

#### The Number of Head Entries in the Juice Dispensing Unit on the Training and Testing Days in the Trace- and Delay-Conditioned Animals

During the testing, both the trace- and delay-conditioned animals showed a significant improvement in the performance of their respective tasks. The learning curve of the trace- and delay-conditioned animals are shown in Figures 1C,D [Compared to the training day, the percent increase in the performance of trace-conditioned animals during different sessions were: session-1: 41.6% increase (Tukey *p* < 0.05); session-2: 11% increase (not significant); session-3: 47.5% increase (Tukey *p* < 0.01); session-4: 38.3% increase (Tukey *p* < 0.01); session-5: 22.6% increase (not significant). The performance of delay-conditioned animals during different sessions were: session-1: 70.1% increase (Tukey *p* < 0.001); session-2: 73.3% increase (Tukey *p* < 0.001); session-3: 65.2% increase (Tukey *p* < 0.01); session-4: 47.5% increase (Tukey *p* < 0.01); session-5: 37% increase (Tukey *p* < 0.05)].

**Figure 2:**
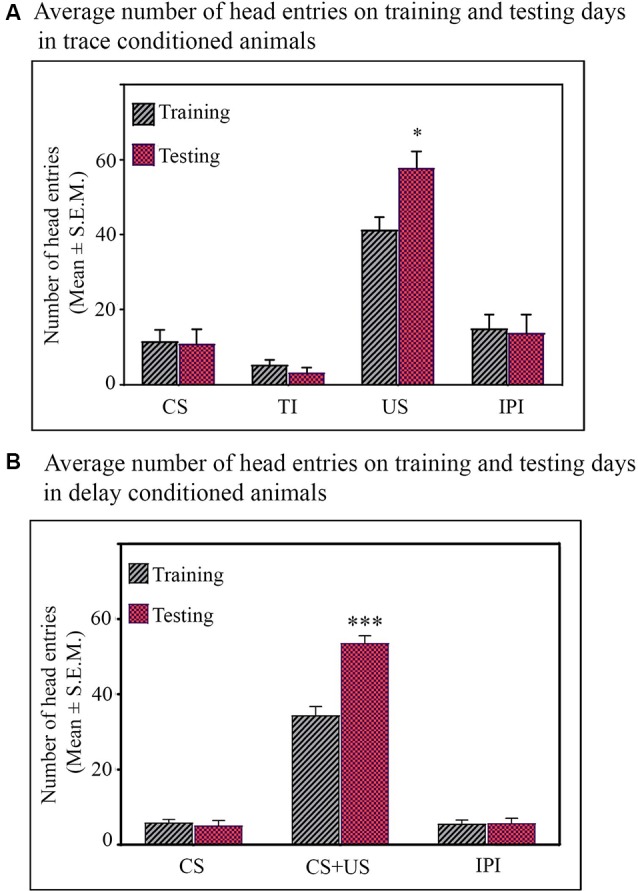
Average number of head entries on the training and testing days in the **(A)** trace-conditioned animals (*n* = 7). Animals showed significantly more number of head entries during the US presentation on testing day (*p < 0.05; *F*_(1,13)_ = 6.73, one-way analysis of variance (ANOVA) followed by Tukey *post hoc*). The number of head entries during CS alone, TI, and IPI were comparable on training and testing days. **(B)** Delay-conditioned animals (*n* = 6). Animals showed significantly more number of head entries during the CS-US paired presentation on the testing day (***p < 0.001; *F*_(1,11)_ = 36.97, one-way ANOVA followed by Tukey *post hoc*). The number of head entries during CS alone and IPI were comparable on the training and testing days.

Further, Tukey *post hoc* comparison revealed that on the testing day, the animals in the trace-conditioned group showed a significant increase in the number of head entries during the US presentation phase compared to the training day. The number of head entries was 39.6% (*P* < 0.05, *F*_(1,13)_ = 6.739) more on the testing day compared to the training day (during training *σ*^2^ = 58.43; during testing *σ*^2^ = 141.15; Cohen’s *d* = 1.45; power = 0.79 at alpha level 0.05). However, the number of head entries in the juice dispensing unit during the CS presentation, TI, and IPI was comparable on the training and testing days (Figure 2A). Similarly, the delay-conditioned animals showed 53.5% increase (*P* < 0.001, *F*_(1,11)_ = 36.97) in number of head entries during the CS-US paired presentation compared to the training day (during training *σ*^2^ = 27.94; during testing *σ*^2^ = 21.74; Cohen’s *d* = 3.51; power = 1.0 at alpha level 0.05). The number of head entries was comparable during CS alone and IPI on the training and testing days (Figure 2B).

#### The Changes in the Number of Proliferating Cells in the DG Area of the Hippocampus After Trace- and Delay-Conditioning

**Figure 3:**
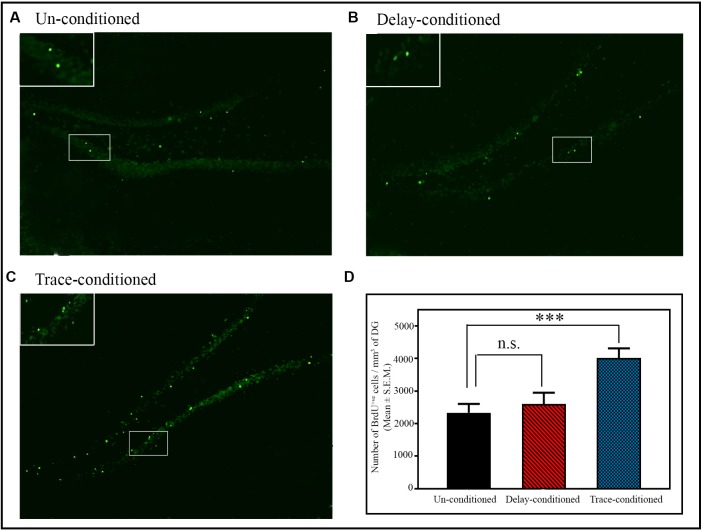
The number of BrdU^+ve^ cells in the dentate gyrus (DG) of the hippocampus after delay- and trace-conditioning. Photomicrographs of the hippocampus (10× magnification) of **(A)** un-conditioned and **(B)** delay-conditioned and **(C)** trace-conditioned animals. Green dots show the BrdU^+ve^ cells. Magnified views of BrdU^+ve^ cells are shown in the inset. **(D)** An average number of BrdU^+ve^ cells/mm^3^ of DG in the un-conditioned (*n* = 6), delay-conditioned (*n* = 6) and trace-conditioned animals (*n* = 7). The BrdU^+ve^ cells significantly increased in the trace-conditioned animals (***p < 0.001; *F*_(2,18)_ = 8.29, one-way ANOVA followed by Tukey *post hoc*), compared to the un-conditioned control animals. The BrdU^+ve^ cells in the delay-conditioned animals were comparable to the un-conditioned control animals. n.s., not-significant.

The number of proliferating cells significantly increased in the DG area of the hippocampus after appetitive trace-conditioning but not after delay-conditioning (Figure 3). Representative photomicrographs of BrdU positive immunostained cells of the un-conditioned, delay-conditioned, and trace-conditioned animals are shown in Figures 3A–C. Animals trained for the appetitive trace-conditioning showed 71.9% increase in BrdU^+ve^ cells/mm^3^ of DG (*p* < 0.001; *F*_(2,18)_ = 8.29) compared to the un-conditioned control animals (Figure 3D; sample variance in un-conditioned group *σ*^2^ = 14,5987.00; trace-conditioned group *σ*^2^ = 75,260.97; Cohen’s *d* = 1.77; power = 0.88 at alpha level 0.05). However, the number of proliferating cells in the DG area of the hippocampus after delay-conditioning did not increase. The number of proliferating cells in the delay-conditioned and un-conditioned control groups was comparable (Figure 3D).

Similar to the changes in the number of BrdU^+ve^ cells, the number of BrdU^+ve^ and Ki67^+ve^ double-labeled cells/mm^3^ of DG also significantly increased in the trace-conditioned animals compared to the un-conditioned animals (*p* < 0.05; *F*_(2,8)_ = 7.73; Supplementary Figure S1). Out of total BrdU^+ve^ cells, a total of 92.30% cells were BrdU^+ve^ and Ki67^+ve^ double-labeled cells in the trace-conditioned animals. Nevertheless, the number of BrdU^+ve^ and Ki67^+ve^ double-labeled cells/mm^3^ of DG in the delay-conditioned group did not change (Supplementary Figure S1). In the trace-conditioned animals, the number of BrdU^+ve^ cells/mm^3^ of DG increased by 68.93% (*p* < 0.05; *F*_(2,8)_ = 5.67; sample variance in un-conditioned group *σ*^2^ = 56,707.12; trace-conditioned group *σ*^2^ = 78,881.74; Cohen’s *d* = 2.10; power = 0.66 at alpha level 0.05), the number of Ki67^+ve^ cells/mm^3^ of DG increased by 69.67% (*p* < 0.05; *F*_(2,8)_ = 6.58; sample variance in un-conditioned group *σ*^2^ = 48,470.01; trace-conditioned group *σ*^2^ = 59,023.50; Cohen’s *d* = 2.26; power = 0.61 at alpha level 0.05), while the number of BrdU^+ve^ and Ki67^+ve^ double-labeled cells/mm^3^ of DG increased by 79.13% (*p* < 0.05; *F*_(2,8)_ = 7.73; sample variance in un-conditioned group *σ*^2^ = 42,916.91; trace-conditioned group *σ*^2^ = 51,754.62; Cohen’s *d* = 2.33; power = 0.75 at alpha level 0.05) compared to the un-conditioned animals (Supplementary Figure S1).

**Figure 4:**
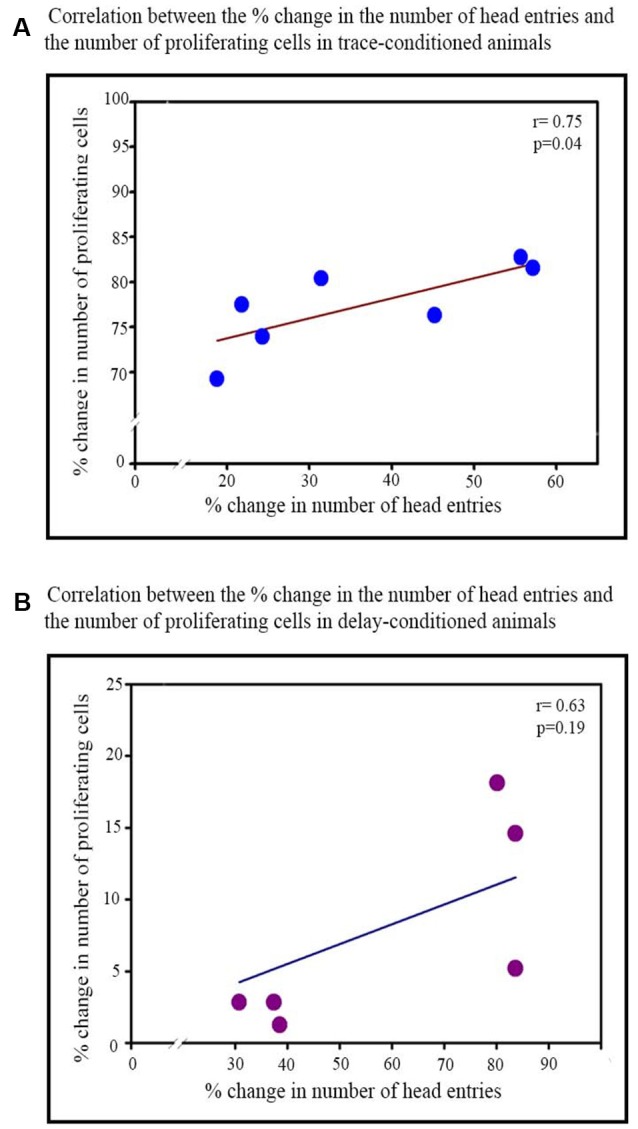
Correlation between the percent increase in the number of head entries on testing day and percent increase in the number of proliferating cells in the trace- and delay-conditioned animals. **(A)** Trace-conditioned animals: the percent increase in the number of proliferating cells positively significantly correlated with the percent increase in the number of head entries on the testing day (*p < 0.05).** (B)** Delay-conditioned animals: the percent increase in the number of proliferating cells did not correlate significantly with the percent increase in the number of head entries on the testing day.

Furthermore, the percent increase in the number of head entries on the testing day significantly positively correlated with the percent increase in the number of proliferating cells in the trace-conditioned animals (*r* = 0.75; *p* < 0.05; Figure 4A). However, the percent increase in the number of head entries in the delay-conditioned animals did not correlate with the percent increase in the number of proliferating cells (*r* = 0.63; *p* > 0.05; Figure 4B).

Besides, we also investigated if the appetitive delay- or trace-conditioning influence cell proliferation in the SVZ (an area outside the hippocampus). We observed that the number of proliferating cells in the SVZ did not change either after delay or trace-conditioning. The number of proliferating cells in the delay- and trace-conditioned animals were comparable with that of the un-conditioned control animals (Supplementary Figure S2). It suggests that hippocampal-dependent appetitive learning does not influence AHN outside the hippocampus area.

#### The Changes in the Number of Cells Progressed Towards the Neuronal Lineage in the DG Area of the Hippocampus After the Trace- and Delay-Conditioning

**Figure 5:**
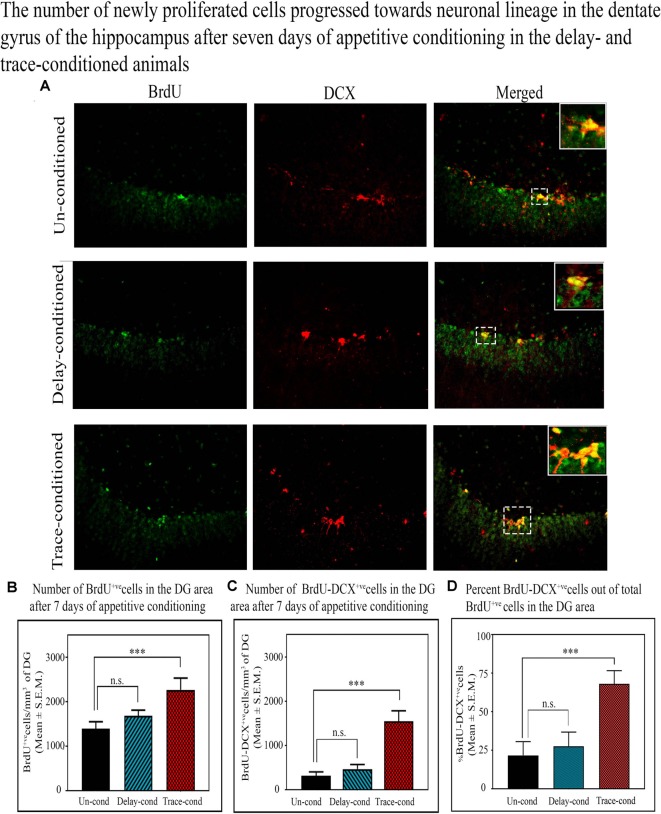
The number of newly proliferated cells progressed towards the neuronal lineage in the DG of the hippocampus after 7 days of appetitive conditioning in the delay- and trace-conditioned animals. **(A)** Photomicrographs (20X magnification) showing BrdU^+ve^, DCX^+ve^, and BrdU^+ve and^ DCX^+ve^ double-labeled cells in the DG of the hippocampus in the un-conditioned, delay-conditioned, and trace-conditioned animals. Green dots show the BrdU^+ve^, red dots show DCX^+ve^, and yellow and orange dots show BrdU^+ve^ & DCX^+ve^ double-labeled cells. Magnified views of BrdU^+ve^, DCX^+ve^, and BrdU^+ve^ & DCX^+ve^ cells are shown in the inset. An average number of **(B)** BrdU^+ve^ cells/mm^3^ of DG **(C)** double-labeled BrdU^+ve and^ DCX^+ve^ cells/mm^3^ of DG in the un-conditioned (*n* = 6), delay-conditioned (*n* = 6) and trace-conditioned (*n* = 6) animals. The BrdU^+ve^ cells significantly increased in the trace-conditioned animals (***p < 0.001; *F*_(2,17)_ = 53.33, one-way ANOVA followed by Tukey *post hoc*), compared to the un-conditioned control animals. The BrdU^+ve^ cells in the delay-conditioned animals were comparable to the un-conditioned control animals. Similarly, the average number of BrdU^+ve and^ DCX^+ve^ double-labeled cells were significantly more in trace-conditioned animals compared to un-conditioned animals (***p < 0.001, *F*_(2,17)_ = 14.90, one-way ANOVA followed by Tukey *post hoc*). **(D)** Out of total BrdU^+ve^ cells, majority cells (67.9%) significantly progressed towards the neuronal lineage in the trace-conditioned animals (***p < 0.001, *F*_(2,17)_ = 27.91, one-way ANOVA followed by Tukey *post hoc*). Whereas, the progression of dividing cells in the delay-conditioned towards neuronal lineage were comparable to the un-conditioned animals. n.s., non-significant.

Doublecortin (DCX) protein is a marker of the immature neurons, and here we observed that the number of BrdU^+ve^ and DCX^+ve^ double-labeled cells in the DG significantly increased only after trace-conditioning (*p* < 0.001, *F*_(2,17)_ = 14.90) but not after delay-conditioning (Figure 5). The number of BrdU^+ve^ labeled cells on the 8th day after trace-conditioning was significantly more compared to un-conditioned animals (Tukey *p* < 0.001; sample variance in un-conditioned group *σ*^2^ = 180,138.64; trace-conditioned group *σ*^2^ = 138,422.26; Cohen’s *d* = 6.05; power = 1.00 at alpha level 0.05; Figure 5B). The number of BrdU^+ve^ labeled cells on the 8th day after delay-conditioned animals did not change (Figure 5B). The number of BrdU^+ve^ and DCX^+ve^ double-labeled cells also significantly increased by 399.3% (Tukey *p* < 0.001) in the trace-conditioned animals compared to the un-conditioned animals (sample variance in un-conditioned group *σ*^2^ = 3724.14; trace-conditioned group *σ*^2^ = 26,830.39; Cohen’s *d* = 7.65; power = 0.98 at alpha level 0.05), but it did not change significantly in the delay-conditioned animals (Figure 5C). Interestingly, out of total BrdU^+ve^ cells, majority cells significantly progressed towards the neuronal lineage in the trace-conditioned animals (*p* < 0.001, *F*_(2,17)_ = 27.91, one-way ANOVA followed by Tukey *post hoc* test). In the trace-conditioned animals, a total of 67.9% cells out of total BrdU^+ve^ cells (Tukey *p* < 0.001) progressed toward neuronal lineage, whereas only 27.5% and 21.7% cells in delay-conditioned and un-conditioned groups progressed toward the neuronal lineage (Figure 5D).

### Experiment-II

#### The Changes in the Expression Level of Phosphorylated and Total of Erk1 and Erk2 Proteins in the TH, DH, and VH After Trace-Conditioning

**Figure 6:**
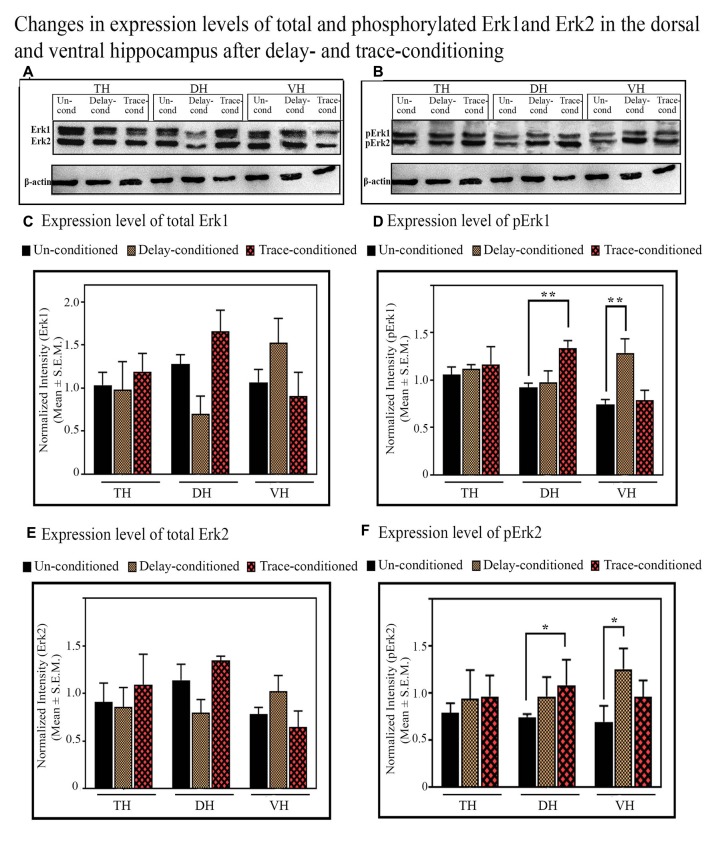
The changes in the expression levels of total and phosphorylated Erk1 and Erk2 proteins after trace- and delay-conditioning in the total hippocampus (TH), dorsal hippocampus (DH) and ventral hippocampus (VH). **(A)** Immunoblots showing bands of Erk1, Erk2, and β-actin (loading control) in TH, DH, and VH. **(B)** Immunoblots showing bands of pErk1, pErk2, and β-actin (loading control) in TH, DH, and VH. The same β-actin blot [as is shown with Erk1, Erk2 in **(A)**] is shown here along with pErk1 and pErk2 blots for comparison. **(C)** No significant difference was observed in the expression level of Erk1 among the groups in TH, DH, and VH. **(D)** The expression level of pErk1 did not change in the TH in the trace- and delay-conditioned animals, however, it significantly increased in the DH in the trace-conditioned animals (**Tukey *p* < 0.01, One way ANOVA; *n* = 6) and in the VH in the delay-conditioned animals (**Tukey *p* < 0.01, One way ANOVA; *n* = 6), compared to the un-conditioned control animals (*n* = 6). **(E)** The expression level of Erk2 did not change significantly in the TH, DH, and VH, in the trace- and delay-conditioned animals compared to the un-conditioned control animals. **(F)** The expression level of pErk2 did not change in the TH, in the trace- and delay-conditioned animals. However, it significantly increased in the DH in the trace-conditioned animals (*n* = 6), and in the VH in the delay-conditioned animals (*n* = 6) compared to the un-conditioned group (*n* = 6; *Tukey *p* < 0.05; One way ANOVA).

In the TH, no significant difference was observed in the expression level of total and phosphorylated Erk1 and Erk2 proteins after trace-conditioning (Figure 6). The expression level of Erk1 and Erk2 proteins also did not change in the DH and VH (Figures 6A,C,E). However, the expression level of pErk1 (*p* < 0.01; *F*_(2,17)_ = 6.95), and pErk2 (*p* < 0.05; *F*_(2,17)_ = 7.25) significantly increased after trace-conditioning in the DH but not in the VH (Figures 6B,D,F). The expression level of pErk1 in DH increased by 44.8% (Tukey *p* < 0.01) and pErk2 increased by 45.9% (Tukey *p* < 0.05) compared to the un-conditioned control animals (Figures 6B,D,F; sample variance of pErk1 in the un-conditioned group *σ*^2^ = 0.06; trace-conditioned group *σ*^2^ = 0.01; Cohen’s *d* = 3.48; power = 0.99 at alpha level 0.05; sample variance of pErk2 in the un-conditioned group *σ*^2^ = 0.01; trace-conditioned group *σ*^2^ = 0.03; Cohen’s *d* = 2.95; power = 0.99 at alpha level 0.05).

#### The Changes in the Expression Level of Phosphorylated and Total of Erk1 and Erk2 Proteins in the TH, DH and VH After Delay-Conditioning

The expression level of Erk1, Erk2, pErk1, and pErk2 did not change in the TH after delay-conditioning (Figure 6). The expression of Erk1, Erk2, pErk1, and pErk2 in the TH were comparable in the delay-conditioned and un-conditioned animals (Figure 6). Similarly, the levels of Erk1, Erk2, pErk1, and pErk2 in the DH were also comparable in the delay-conditioned and un-conditioned animals (Figure 6). In the VH, however, the delay-conditioned animals showed a significant increase in the expression of pErk1 (*p* < 0.01;*F*_(2,17)_ = 7.65) and pErk2 (*p* < 0.05; *F*_(2,17)_ = 6.07) proteins compared to the un-conditioned control group. The expression level of pErk1 increased by 72.8% (Tukey *p* < 0.01), whereas the level of pErk2 increased by 81.8% (Tukey *p* < 0.05) compared to the un-conditioned control animals (Figures 6B,D,F; sample variance of pErk1 in un-conditioned group *σ*^2^ = 0.15; delay-conditioned group *σ*^2^ = 0.08; Cohen’s *d* = 2.73; power = 0.99 at alpha level 0.05; sample variance of pErk2 in un-conditioned group *σ*^2^ = 0.02; delay-conditioned group *σ*^2^ = 0.05; Cohen’s *d* = 2.75; power = 0.99 at alpha level 0.05). Nevertheless, the expression level of Erk1 and Erk2 in the VH did not change after delay-conditioning (Figures 6A,C,E).

#### The Changes in the Expression of CREB and pCREB in the TH, DH, and VH After Trace-Conditioning

**Figure 7:**
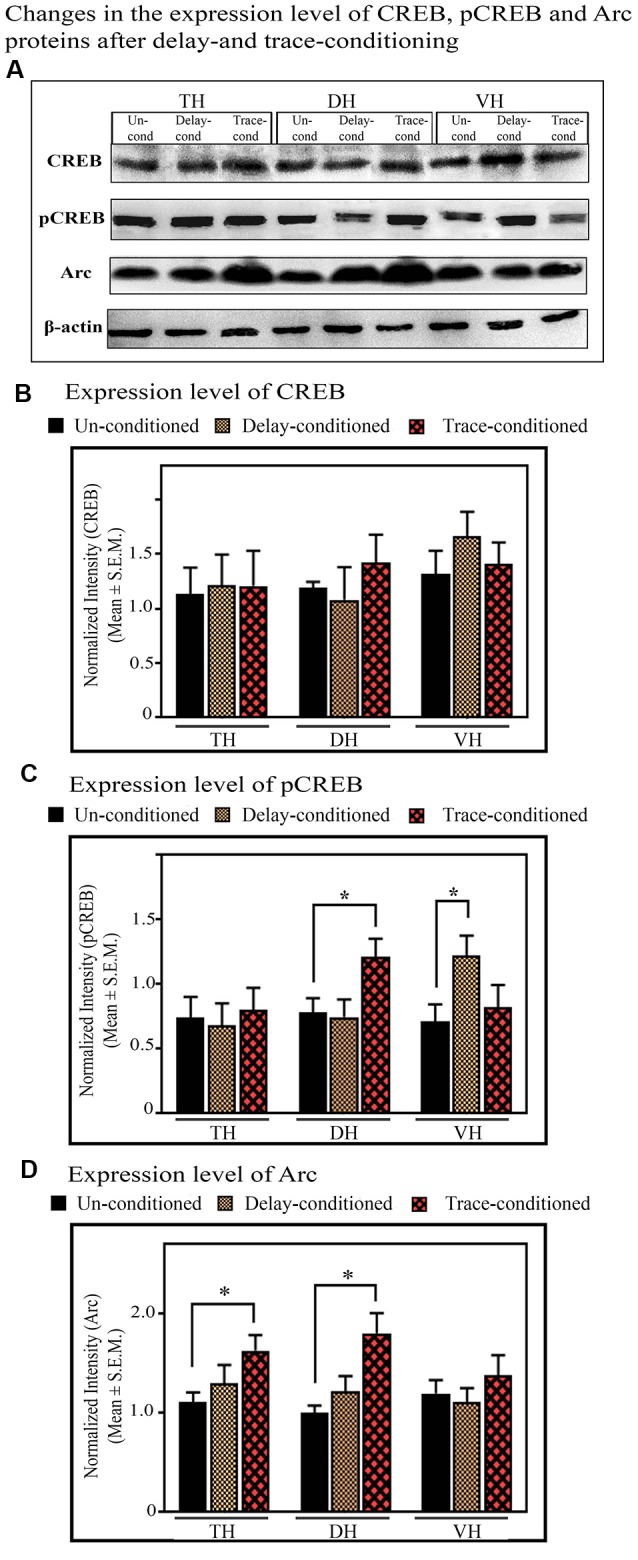
The changes in the expression levels of CREB, pCREB, and Arc proteins after trace- and delay-conditioning in the total hippocampus (TH), DH, and VH. **(A)** Immunoblots showing bands of CREB, pCREB, Arc, and β-actin (loading control) in the TH, DH, and VH. The same β-actin blot, as is represented in Figure 6 is shown here for comparison. **(B)** The expression level of CREB was comparable in the un-conditioned control group (*n* = 6), trace- (*n* = 6), and delay- (*n* = 6) conditioned group in TH, DH, and VH. **(C)** The trace- (*n* = 6) and delay- (*n* = 6) conditioned animals showed significantly enhanced pCREB levels in the DH and VH, respectively, compared to the un-conditioned (*n* = 6) animals (*Tukey *p* < 0.05, One way ANOVA). In the TH, no significant difference was observed between the groups. **(D)** The trace-conditioned animals (*n* = 6) showed significantly increased Arc expression levels in both TH and DH (*Tukey *p* < 0.05, One way ANOVA), compared to the un-conditioned group (*n* = 6). The expression of Arc in the VH was comparable between groups.

The expression level of CREB and pCREB did not change in the TH after trace-conditioning. The expression levels of both CREB and pCREB proteins in the TH were comparable in both the trace- and un-conditioned animals (Figures 7A–C). The levels of CREB in the DH and VH were also comparable in both, the trace- and un-conditioned animals (Figures 7A,B). In the DH, however, the trace-conditioned animals showed a significant increase in the expression of pCREB protein (*p* < 0.05; *F*_(2,17)_ = 4.88) compared to the un-conditioned control group. The expression level of pCREB increased by 55.2% (Tukey *p* < 0.05) compared to the un-conditioned control animals (Figure 7C; sample variance of pCREB in un-conditioned group *σ*^2^ = 0.01; trace-conditioned group *σ*^2^ = 0.06; Cohen’s *d* = 2.74; power = 0.93 at alpha level 0.05). Nevertheless, the expression level of pCREB in the VH did not change after trace-conditioning (Figure 7C).

#### The Changes in the Expression of CREB and pCREB in the TH, DH, and VH After Delay-Conditioning

The expression level of CREB and pCREB did not change in the TH after delay-conditioning. The expression of CREB and pCREB in the TH were comparable in both the delay- and un-conditioned animals (Figures 7A–C). Similarly, the levels of CREB and pCREB in the DH were also comparable in the delay- and un-conditioned animals (Figures 7A–C). In the VH, however, the delay-conditioned animals showed a significant increase in the expression of pCREB (*p* < 0.05; *F*_(2,17)_ = 5.31) compared to the un-conditioned control group. The expression level of pCREB increased by 71.94% (Tukey *p* < 0.05) compared to the un-conditioned control animals (Figure 7C; sample variance of pCREB in the un-conditioned group *σ*^2^ = 0.04; delay-conditioned group *σ*^2^ = 0.02; Cohen’s *d* = 3.01; power = 0.99 at alpha level 0.05). Nevertheless, the expression level of CREB in the VH did not change after delay-conditioning (Figure 7B).

#### The Changes in the Expression of Arc in the TH, DH, and VH After Trace-Conditioning

In the TH, the expression level of Arc protein significantly increased (*p* < 0.05, *F*_(2,17)_ = 5.06) after trace-conditioning. It increased by 47.16% (Tukey *p* < 0.05) compared to the un-conditioned control (Figures 7A,D; sample variance of Arc protein in un-conditioned group *σ*^2^ = 0.03; trace-conditioned group *σ*^2^ = 0.07; Cohen’s *d* = 2.32; power = 0.96 at alpha level 0.05). The trace-conditioned animals also showed a significant increase in the expression level of Arc protein in the DH (*p* < 0.05, *F*_(2,17)_ = 5.28). The expression level of Arc protein increased by 79.75% (Tukey *p* < 0.05) compared to the un-conditioned control group (Figures 7A,D; sample variance of Arc protein in un-conditioned group *σ*^2^ = 0.02; trace-conditioned group *σ*^2^ = 0.12; Cohen’s *d* = 2.12; power = 0.93 at alpha level 0.05). The expression level of Arc protein did not change in the VH in the trace-conditioned group (Figures 7A,D).

#### The Changes in the Expression of Arc in the TH, DH, and VH After Delay-Conditioning

No significant difference was observed in Arc expression in TH, DH or VH, after delay-conditioning. The level of Arc protein in the TH, DH, and VH were comparable to the un-conditioned group (Figures 7A,D).

## Discussion

In Experiment-I, we observed that appetitive trace- but not delay-conditioning task augmented the number of proliferating cells in the DG. Further, we observed that the majority of these proliferative cells progressed towards the neuronal lineage only in the trace-conditioned animals but not in the delay-conditioned animals. The nature of appetitive conditioning primarily depends upon the temporal pattern of CS and US appearance during the trials. In trace-conditioning, there is a small-time lag, “the trace interval” between the offset and onset of CS and US, which makes it hippocampus-dependent (Chowdhury et al., 2011; Tripathi et al., 2019). Various reports have demonstrated the role of the hippocampus in the formation of the temporal association between the CS and US in trace memory (Christian and Thompson, 2003). On the other hand, the CS-US association forms outside the hippocampus in delay-appetitive-conditioning (Wallenstein et al., 1998; Rodriguez and Levy, 2001; Bangasser et al., 2006; Woodruff-Pak and Disterhoft, 2008; Moustafa et al., 2013). It has also been reported that only hippocampus-dependent tasks potentiate the neuronal proliferation and/or survival in the DG (Döbrössy et al., 2003; Deng et al., 2009, 2010). The hippocampus-independent tasks, however, do not affect neurogenesis (Sisti et al., 2007; Deng et al., 2010; Abrous and Wojtowicz, 2015; Gonçalves et al., 2016). Thus, our data that the number of newly generated neurons in the DG area increased only after trace-conditioning but not after delay-conditioning is in agreement with these previous findings. Further, we did not observe any change in the number of proliferating cells in the SVZ after trace-conditioning. It suggested that the hippocampal-dependent trace-appetitive conditioning explicitly influences AHN in the DG area of the hippocampus. It does not influence neurogenesis outside the hippocampus area.

Several previous reports have shown that the number of newborn cells in the DG significantly increased after learning spatial learning task (Morris water maze) and conditioned learning tasks such as trace-eyeblink-conditioning, contextual fear-conditioning, et cetera (Gould et al., 1999; Hairston et al., 2005; Olariu et al., 2005). However, it has also been found in some studies that spatial learning or conditioning may not influence or somewhat decrease the number of proliferating cells in the DG area of the hippocampus (Döbrössy et al., 2003; Ambrogini et al., 2004; Olariu et al., 2005). In these learning paradigms, animals usually experience stressful or life-threatening situations during the training. In this study, we have used an appetitive conditioning paradigm, where anxiogenic or fear-associated factors are not at all present during the training; instead, a strong motivational component contributes to learning. It has consistently been found that enriched motivational environment always augments cell proliferation in the DG area of the hippocampus (Speisman et al., 2013; Monteiro et al., 2014). Therefore, the differences in the results can be attributed to the experience of positive emotional stimulus and/or stressful or life-threatening factors during the training. Appetitive conditioned learning plays a crucial role in the survival of the organism under its natural habitat. It influences the approach behavior of the animals for food in a hostile or conducive environment. Animals discriminate between safe and unsafe conditions and accordingly determine whether to approach the food or not. Thus it ultimately helps them to develop life-saving skills (Tripathi et al., 2019). Also, the food-storing birds exhibit seasonal changes in the hippocampus size and food-storing behavior. Intriguingly, very high rates of neurogenesis have been observed in the food storing birds. The increased neurogenesis in the hippocampus in food-storing birds is attributed to the cognitive involvement of the hippocampus in storing and retrieving food (Barnea and Pravosudov, 2011). These findings, along with our results, suggest that learning skills through appetitive trace-conditioning task augment neurogenesis in the hippocampus. Further, the long-distance traveling migratory birds are comparatively exposed to more diverse spatial cues during their course of journey compared to short-distance traveling migratory birds. Interestingly, a significant positive correlation has been observed between the number of newly generated neuronal cells in the hippocampus and migratory distances (Barkan et al., 2016). We have also observed in this study that the trace-conditioned animals exhibited a significant positive correlation between the number of proliferating cells and the extent of learning (number of head entries). It, thus, suggests that learning-induced recruitment of new neuronal cells may help in better estimation of their surroundings and memory retrieval.

Learning-induced neuronal activity and periodic replay facilitate memory consolidation as well as adult neurogenesis. The expression of Arc changes along with neuronal activity and hence used as a marker of neuronal activity and different forms of synaptic plasticity. In our study, we have observed that the expression of Arc protein significantly increased in the trace-conditioned animals. These results suggest that neuronal activity in the DH increased after trace-conditioning. Previously a marked increase in hippocampal neuronal activity has been reported after the trace-conditioning (McEchron and Disterhoft, 1999; Weible et al., 2006). The increased neural activation in response to the external stimuli such as the behavioral experiences may result in increased expression of activity-regulated cytoskeleton protein (Arc), which is also crucial for neural encoding and synaptic plasticity (Lyford et al., 1995; Guzowski et al., 1999, 2001; Uebele et al., 2009; Das et al., 2018; Janz et al., 2018; Nikolaienko et al., 2018). In the present study, a significant increase in the expression of Arc protein in the entire hippocampus after trace-appetitive-conditioning could be attributed to the trace-conditioning induced hippocampal activation. The increased neuronal activity after trace-conditioning may, in turn, trigger the neuronal proliferation in the adult DG. In delay-conditioned animals, the expression level of Arc remained unchanged, and also, the number of proliferating cells was unaltered. It could be attributed to the fact that the hippocampal neuronal activity may not increase in the delay-conditioning. Interestingly, we find that the expression level of Arc protein selectively increased in the DH, but not in VH, after trace-conditioned training, which suggests the active participation of DH in the appetitive trace-conditioning.

Based on the learning paradigms, many theories have been proposed, over the years, regarding the functional dissociation of the hippocampus along its dorsoventral axis (Moser et al., 1993, 1995; Moser and Moser, 1999; Bannerman et al., 2004; Fanselow and Dong, 2010). The DH plays a predominant role in hippocampus-dependent tasks such as contextual fear-conditioning, spatial learning, trace-eyeblink-conditioning, etc. The appetitive conditioning is also altered with total inactivation of DH neuronal activity (Pezze et al., 2018), suggesting that the DH plays a crucial role in the appetitive conditioning. In our study, the induced expressions of Arc, pCREB, pErk1, and pErk2 proteins in the DH, after trace-conditioning is in agreement with this concept. However, Thibaudeau et al. (2007) have found that the DH lesion did not affect the acquisition of appetitive trace-conditioned learning. Longer and shorter trace intervals between the CS and US also play an essential role in learning (Chowdhury et al., 2011). Thibaudeau et al. (2007) have used shorter (2 s) trace interval, whereas, we and others have used the longer trace interval. Possibly the DH may be playing a role in the temporal association between the CS and US. The shorter trace interval could be hippocampal-in-dependent, whereas longer trace interval may require the DH for memory consolidation. Reports also suggest that the VH plays a modulatory role in emotional as well as the hippocampal-independent learning task, for example, reward learning, delay-fear-conditioning, et cetera (Moser et al., 1993; Yoon and Otto, 2007; Esclassan et al., 2009; Kheirbek and Hen, 2011; Kheirbek et al., 2013). The VH but not the DH lesion affects the consolidation of cued fear memory (Esclassan et al., 2009). Also, the DH lesion-induced deficit in the trace-conditioned memory having the long-trace interval but not the short-trace interval (Chowdhury et al., 2005). In our results, the increased expression of pErk1, pErk2, and pCREB in the VH could be associated with its unique role in processing the information associated with delay-conditioning. These results are in favor of the functional dissociation of the hippocampus, reported about the trace- and delay-fear-conditioning (Yoon and Otto, 2007; Esclassan et al., 2009). No change in the expression/activation level of CREB, Erk1 and Erk2 proteins in the TH in trace/delay conditioned animals, compared to that of un-conditioned control, can be further associated with the possibility of functional division within the hippocampus as per the demand of the tasks.

In the adult brain, the expansion of the neural population and ultimate incorporation of these neural cells is exquisitely controlled by molecular/genetic networks (Dworkin and Mantamadiotis, 2010). Transcription factors are the key regulators in orchestrating the cell-specific temporal expression of factors involved in these signaling networks. In this regard, MAPK/Erk has been reported as a rheostat to influence neurogenesis (Vithayathil et al., 2015; Krawczyk et al., 2019). Activated Erk1 and Erk2 may lead to diverse cellular responses, such as protein synthesis, cell proliferation, and survival, et cetera (Garcia et al., 2002; Lee et al., 2016). It was shown that activation of Erk1 and Erk2, subsequently activated its downstream target cAMP response element-binding protein (CREB), which resulted in increased neurogenesis (Lim et al., 2014). Thus, in the present finding, increased pErk1, and pErk2 levels in trace-conditioned animals in the DH could be a possible regulator of increased cell-proliferation in adult DG after appetitive trace-conditioning.

Further, the role of CREB (a downstream target of MAPK/Erk pathway) has been widely investigated as a possible regulator of neurogenesis (Nakagawa et al., 2002; Fujioka et al., 2004; Merz et al., 2011). Nakagawa et al. (2002) in their study showed that stabilized CREB and its phosphorylation are essential for increased neuronal proliferation in the DG. Also, in a mouse model of Alzheimer’s disease, increased CREB expression in the DH rescued spatial memory impairments (Yiu et al., 2011). In the present investigation, significant up-regulation of pCREB in the DH after the appetitive trace-conditioning further supports the role of CREB in the appetitive trace-conditioned memory and trace-memory mediated increase in cell-proliferation. Since, we did not observe any significant change in the expression of CREB or pCREB in the TH or VH after trace-conditioning, which further strengthens the role of DH as a possible regulator in inducing cell-proliferation in the DG area of the hippocampus in the trace-conditioned animals.

In summary, our results suggest that trace-appetitive-conditioning increases hippocampal cell proliferation and neurogenesis. The up-regulation of Arc protein in the TH and DH, demonstrate the selective activation of DH after trace-conditioning, which could be involved in memory consolidation as well as memory associated increase in cell-proliferation. Besides, the increased expression of pErk1, pErk2, and pCREB proteins in the DH after trace-conditioning and pErk1, pErk2, and pCREB proteins in VH, after delay-conditioning, suggest their active participation in the trace- and delay-conditioning, respectively. It is, however, not known if the expression and activation of Arc, Erk, and CREB proteins in the hippocampus play an instructive or facilitatory role in neurogenesis. Also, it is not clear what is the precise role of these newly proliferated neurons in hippocampal circuit reorganization, underlying trace-memory consolidation. Answers of these questions await future studies. Nevertheless, our results demonstrate that the appetitive trace-conditioning increased cell proliferation and neurogenesis in the adult DG, possibly through the increased activation of Erk and CREB proteins in the DH only.

## Data Availability Statement

The datasets generated for this study are available on request to the corresponding author.

## Ethics Statement

The animal study was reviewed and approved by The Institutional Animal Ethics Committee (IAEC), Jawaharlal Nehru University, New Delhi.

## Author Contributions

ST: performed experiments, generated and analyzed data, and also prepared the manuscript. AV: also performed experiments, generated and analyzed data. SJ: conceived the idea and designed the work, analyzed the data and finalized the manuscript.

## Conflict of Interest

The authors declare that the research was conducted in the absence of any commercial or financial relationships that could be construed as a potential conflict of interest.

## Supplementary Material

FIGURE S1 The changes in the number of BrdU^+ve^ and Ki67^+ve^ double-labeled cells in the DG area in the delay- and trace-conditioned animals (*n* = 9). **(A)** Photomicrographs of DG area showing BrdU^+ve^, Ki67^+ve^, and BrdU^+ve^ and Ki67^+ve^ double-labeled cells in un-conditioned, delay-conditioned, and trace-conditioned animals (10× magnification). Green dots show BrdU^+ve^, red dots show Ki67^+ve^, and yellow and orange dots show BrdU^+ve^ and Ki67^+ve^ double-labeled cells in the DG area of the hippocampus. Bar graphs showing the number of **(B)** BrdU^+ve^ cells/mm^3^ of DG, **(C)** Ki67^+ve^ cells/mm^3^ of DG, and **(D)** BrdU^+ve^ and Ki67^+ve^ double-labeled cells/mm^3^ of DG in un-conditioned (*n* = 3), delay-conditioned (*n* = 3) and trace-conditioned animals (*n* = 3). There was significant increase in the number of BrdU^+ve^ cells (**p* < 0.05; *F*_(2,8)_ = 5.67), Ki67^+ve^ cells (**p* < 0.05; *F*_(2,8)_ = 6.58), and BrdU^+ve^ and Ki67^+ve^ double-labeled cells (**p* < 0.05; *F*_(2,8)_ = 7.73) in the trace-conditioned animals (one-way ANOVA followed by Tukey *post hoc*), compared to the un-conditioned control animals. The numbers of BrdU^+ve^ cells, Ki67^+ve^ cells and BrdU^+ve and^ Ki67^+ve^ double-labeled cells in the delay-conditioned animals were comparable to the un-conditioned control animals. n.s., non-significant.

FIGURE S2 Number of BrdU^+ve^ cells in the SVZ of the trace- (*n* = 3), delay- (*n* = 3), and un-conditioned (*n* = 3) animals. BrdU^+ve^ cells are shown as green dots in histological photomicrographs (10×) of **(A)** un-conditioned, **(B)** trace-conditioned, and **(C)** delay-conditioned animals. **(D)** The number of BrdU^+ve^ cells/mm^3^ area did not change significantly in the SVZ after delay- and trace-conditioning.
