# Supplementary figures and images for: Training on an Appetitive Trace-Conditioning Task Increases Adult Hippocampal Neurogenesis and the Expression of Arc, Erk and CREB Proteins in the Dorsal Hippocampus
Source: Front Cell Neurosci. 2020 Apr 17;14:89. doi: 10.3389/fncel.2020.00089 (PMC7181388; doi:10.3389/fncel.2020.00089)

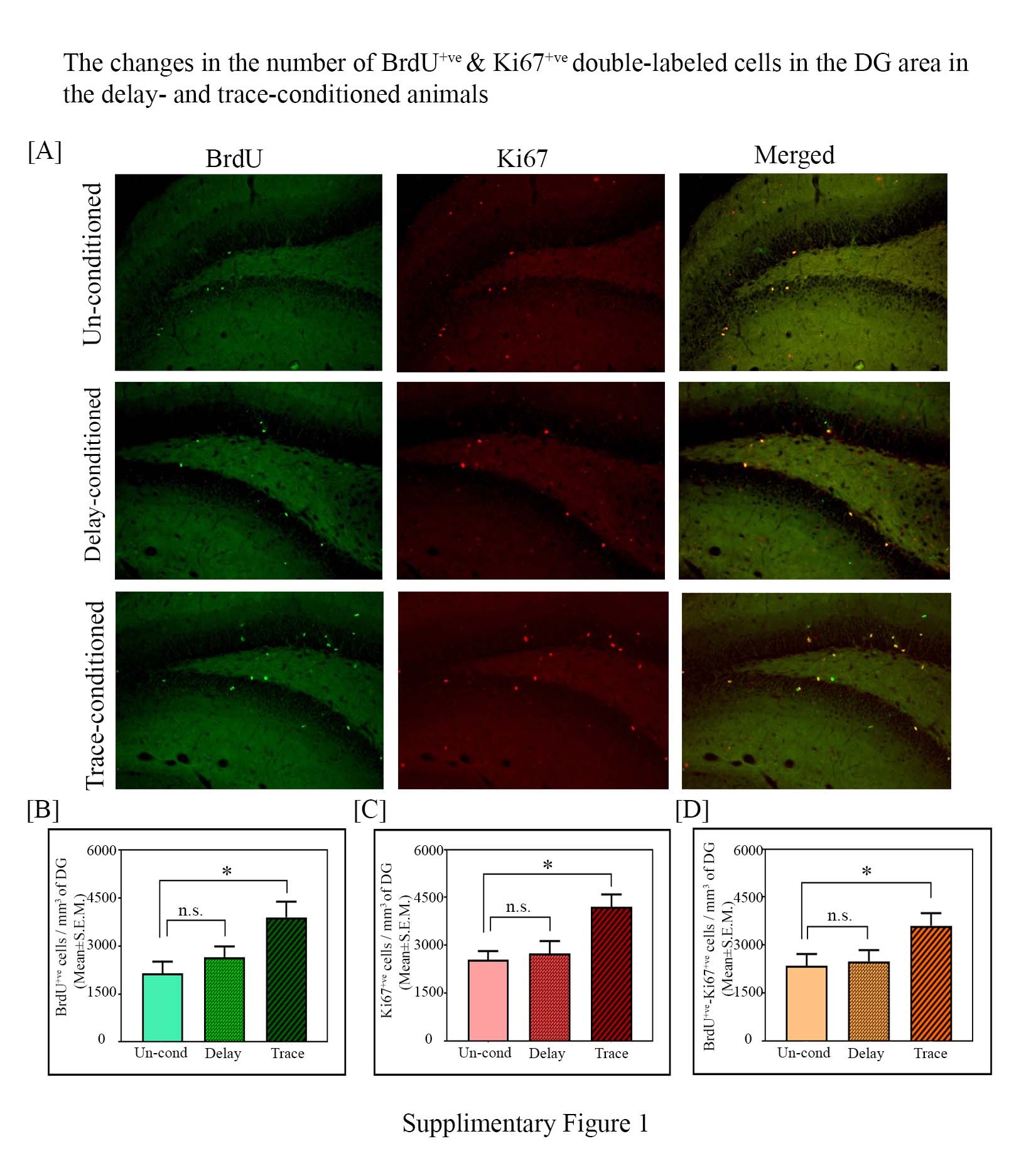

Supplement: FIGURE S1 — The changes in the number of BrdU+ve and Ki67+ve double-labeled cells in the DG area in the delay- and trace-conditioned animals (n = 9). (A) Photomicrographs of DG area showing BrdU+ve, Ki67+ve, and BrdU+ve and Ki67+ve double-labeled cells in un-conditioned, delay-conditioned, and trace-conditioned animals (10× magnification). Green dots show BrdU+ve, red dots show Ki67+ve, and yellow and orange dots show BrdU+ve and Ki67+ve double-labeled cells in the DG area of the hippocampus. Bar graphs showing the number of (B) BrdU+ve cells/mm3 of DG, (C) Ki67+ve cells/mm3 of DG, and (D) BrdU+ve and Ki67+ve double-labeled cells/mm3 of DG in un-conditioned (n = 3), delay-conditioned (n = 3) and trace-conditioned animals (n = 3). There was significant increase in the number of BrdU+ve cells (*p < 0.05; F(2,8) = 5.67), Ki67+ve cells (*p < 0.05; F(2,8) = 6.58), and BrdU+ve and Ki67+ve double-labeled cells (*p < 0.05; F(2,8) = 7.73) in the trace-conditioned animals (one-way ANOVA followed by Tukey post hoc), compared to the un-conditioned control animals. The numbers of BrdU+ve cells, Ki67+ve cells and BrdU+ve and Ki67+ve double-labeled cells in the delay-conditioned animals were comparable to the un-conditioned control animals. n.s., non-significant. [file Image_1.JPEG]

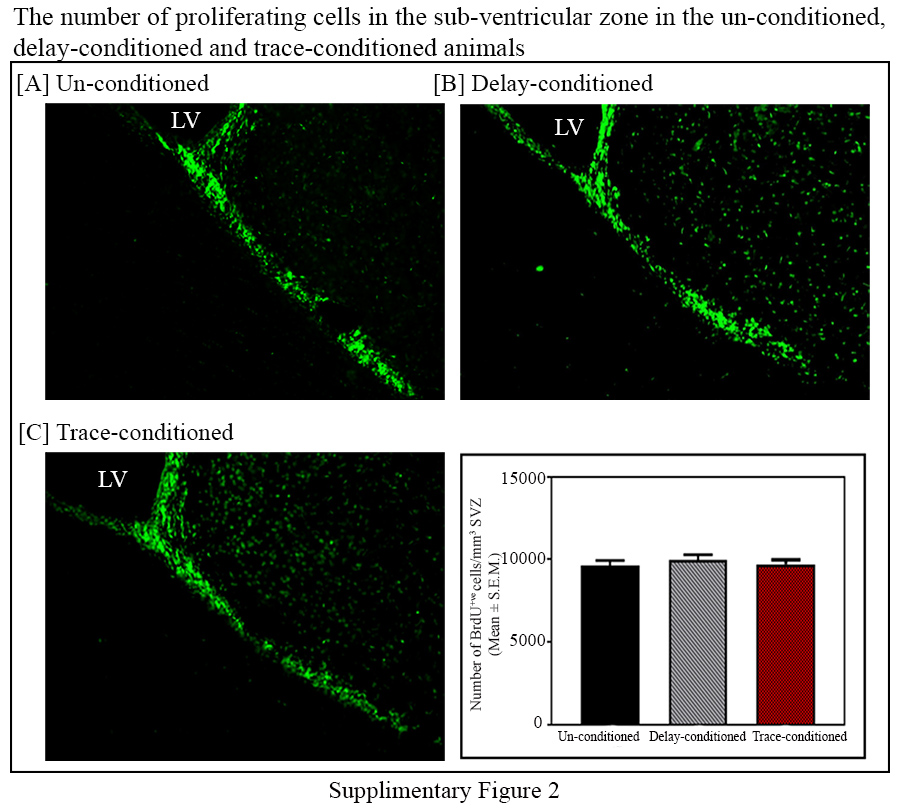

Supplement: FIGURE S2 — Number of BrdU+ve cells in the SVZ of the trace- (n = 3), delay- (n = 3), and un-conditioned (n = 3) animals. BrdU+ve cells are shown as green dots in histological photomicrographs (10×) of (A) un-conditioned, (B) trace-conditioned, and (C) delay-conditioned animals. (D) The number of BrdU+ve cells/mm3 area did not change significantly in the SVZ after delay- and trace-conditioning. [file Image_2.JPEG]
